# Supracricoid partial laryngectomy with cricohyoidoepiglottopexy in patients with radiation therapy failure

**DOI:** 10.1186/1477-7819-7-101

**Published:** 2009-12-19

**Authors:** Kuauhyama Luna-Ortiz, Philippe Pasche, Mario Tamez-Velarde, Veronica Villavicencio-Valencia

**Affiliations:** 1 Department of Head and Neck Surgery, Instituto Nacional de Cancerología (Mexico), Av. San Fernando #22, Tlalpan, Mexico, D.F., 14080, Mexico; 2 Universidad Nacional Autonoma de México (UNAM), Mexico, D.F., Mexico; 3 Service dÒRL, Centre Hospitalier Universitaire Vaudios, Lausanne, Switzerland

## Abstract

**Background:**

To assess functional results, complications, and success of larynx preservation in patients with recurrent squamous cell carcinoma after radiotherapy.

**Methods:**

From a database of 40 patients who underwent supracricoid partial laryngectomy (SCPL) with cricohyoidoepiglottopexy (CHEP) from June 2001 to April 2006, eight patients were treated previously with radiotherapy due to squamous cell carcinoma of the glottic region and were treated for recurrence at the site of the primary cancer.

**Results:**

SCPL with CHEP was performed in six men and two women with a mean age of 67 years due to recurrence and/or persistence at a mean time of 30 months postradiotherapy (in case #8 after concomitant chemoradiotherapy). Bilateral neck dissection at levels II-V was performed in six patients. Only case #8 presented metastasis in one node. In case #5, Delphian node was positive. It was possible to preserve both arytenoids in five cases. Definitive surgical margins were negative. Complications were encountered in seven patients. Follow-up was on average 44 months (range: 20-67 months). Organ preservation in this series was 75%, and local control was 87%. Overall 5-year survival was 50%.

**Conclusions:**

In selected patient with persistence and/or recurrence after radiotherapy due to cancer of the larynx, SCPL with CHEP seems to be feasible with acceptable local control and toxicity. Complications may occur as in previously non-irradiated patients. These complications must be treated conservatively to avoid altering laryngeal function.

## Introduction

Primary radiotherapy as treatment for early cancer of the glottis has been the most used treatment modality due to its low morbidity and excellent prognosis [1,2]. Rate of recurrence is reported to be from 13 to 36% [3-6]. In Mexico, this type of treatment is not an exception because it constitutes the main treatment modality in most oncological centers. However, according to a review of the literature there is only one series published in our country by Rodríguez Cuevas et al. [7], which demonstrates recurrence but does not accurately reflect the recurrence of glottic carcinoma after radiotherapy in Mexican patients. Total laryngectomy continues to be the most frequently used procedure with postradiotherapy recurrence. Several attempts have been made at organ preservation such as the use of laser endoscopic procedures [8,9] or converting vertical hemilaryngectomies [10] to supracricoid laryngectomies [7,11]. The present study was designed to assess functional results, complications, and success in preserving the larynx in patients with recurrent postradiotherapy squamous cell carcinoma.

## Materials and methods

From a database of 40 patients who underwent supracricoid partial laryngectomy (SCPL) with cricohyoidoepiglottopexy (CHEP) from June 2001 to September 2008, eight patients had previously been treated with radiotherapy due to squamous cell carcinoma of the glottic region and sought treatment due to recurrence at the primary site, which would comply with the classic criteria described by Laccourreye et al. [12] for this surgery as follows: when there is less mobility of the arytenoids and subglottic invasion ≤ .5 cm to the posterior commissure. All patients were staged at recurrence. Patients had chest X-rays, computerized tomography of the larynx and neck, nasofibrolaryngoscopy (with biopsy when possible), and suspension microlaryngoscopy to confirm recurrence when nasofibrolaryngoscopy results were inconclusive. In six cases, a bilateral neck dissection at levels II-V was performed, the Delphian node was intentionally searched for, and surgical margins were intra-operatively assessed. Hospital stay was assessed in days, along with the permanence of the tracheostomy and the nasogastric feeding tube for postoperative evolution, but not quality of voice. Demographic data were analyzed using statistical package SPSS for Windows (v.15). Kaplan-Meier method was used to calculate overall survival.

## Results

SCPL with CHEP was performed in six men and two women (mean age: 67 years, median 65 years) due to recurrence and/or persistence of laryngeal cancer (mean time 30 months, median 12 months, postradiotherapy). In case #8, concomitant chemoradiotherapy was used. However, radiotherapy doses were only able to be accurately established in four cases because the remaining cases were referred from other institutions. Bilateral neck dissection at levels II-V was performed in six patients. Only case #8 presented metastasis in one node and case #5 was positive for Delphian node. In five cases it was possible to preserve both arytenoids. Surgical margins were intra-operatively assessed in all cases and, when these were close to being positive, in some cases the margin was widened to include one arytenoid. Definitive surgical margins were negative (Table 1).

**Table 1:** Demographic data and status

Case #	Age	Gender	RT (Gy)	Recurrence, persistence or relapse (months)	Neck dissection	Delphian node	Preserved arytenoids	Status/follow-up (months)
1	56	M	76	11	Bilateral	Negative	2	AwoD (57)
2	43	M	?	144	Bilateral	Negative	2	AwoD (54)
3	70	M	70	36	Bilateral	Negative	2	AwoD (55)
4	65	M	?	5	No	Negative	2	AwoD (54)
5	87	F	?	12	No	Positive	1	AwoD (45)
6	77	M	?	14	Bilateral	Negative	2	LwoD (1)
7	80	F	66	12	Bilateral	Negative	1	LwoD (1)
8	61	M	70	4	Bilateral	Negative	1	AwD (9)

AwoD, Alive without disease; LwoD, Lost without disease; AwD, Alive with disease; RT, radiation therapy.

Table 2 shows postoperative evolution and complications. Complications occurred in seven patients, four with edema of the arytenoids. In case #3, in whom this occurred, resection of the mucosa of the arytenoids was performed, leading to their fixation that induced ankylosis of the arytenoids. Hence, the patient was confined to gastrostomy for 2 years due to chronic aspiration and underwent phoniatric rehabilitation. At 4 1/2 years after treatment, the patient is currently without gastrostomy. Based on this event, subsequent patients with the same complication have been treated only with steroids, leading to better results. Another complication was infection of the tracheostomy in two patients who were treated only with antibiotics. The most severe complication was rupture of the pexy in case #5 on postoperative day 15, necessitating total laryngectomy.

**Table 2:** Postoperative success and complications of SCPL with CHEP in post-radiotherapy recurrences.

Reference (no. of cases)	Mean decannulation (range) days	Mean deglution (range) days	Mean hospital stay (range) days	No. of complications	Comments
(12)[11]	15 (3-30)	30 days in 6 patients	-----	Arytenoid edema (5) Laryngeal stenosis (2) Perichondritis (2) Neck abscess (2) Aspiration pneumonia (1)	Five patients with temporal swallowing difficulties, PEG for 2-6 months

(45, CHEP 15)[14]	---	12	-----	Failure of decannulation (6). Perichondritis and permanent stenosis (2).	

(23)[16]	24	21 (9-45)	26	Aspiration pneumonia (4). Partial necrosis of pexy (1). Cutaneous necrosis (1)	Four patients (17%) with significant swallowing problems, one patient with NTF for 96 days. One patient PEG. Two died due to aspiration pneumonia.

(9, CHEP 6)[18]	11	27	34	Partial RP (1) CWI (2) Fistula and CWI(1) Seroma (1)	One patient was decannulated during hospital admission but a tracheotomy was repeated 3 months after surgery due to edema of laryngeal mucosa. The patient died 15 days later as a consequence of a massive cervical hemorrhage secondary to the erosion of the brachiocephalic artery

(21, CHEP 4)[19]	8.5		30	Abscess & P (1) GI bleeding (1)	One patient died at 9 days due to GI bleeding and AMI

(8)Current series	16 (3-56)	16 (3-60)	10 (7-19)	Arytenoid edema (4), Tracheostomy infection (2), RP (1)	1 patient required PEG for 2 years due to aspiration. Total laryngectomy due to RP

Modified from Motamed M. et al., *Laryngoscope *2006;116:451-5. SCPL = supracricoid partial laryngectomy; CHEP = cricohyoidoepiglottopexy, PEG = percutaneous endoscopy gastrostomy; NFT = nasogastric feeding tube; AMI = acute myocardial infection; GI = gastrointestinal; CWI = cervical wound infection, RP = Rupture of the pexy.

**Figure 1:**
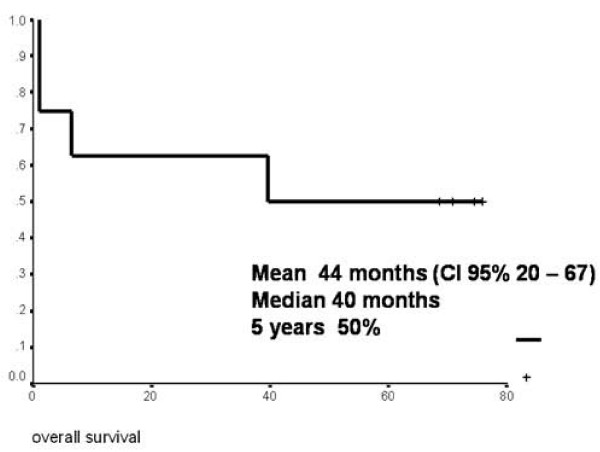
**Overall survival according to Kaplan-Meier**.

Initial TNM classification, as well as that of postradiotherapy recurrence, depicts migration of the stages at the time of recurrence (Table 3). Currently, four patients are alive and disease free, two are alive with pulmonary metastatic disease, and two patients were lost, being disease free with an average follow-up of 44 months, median 45 months (range: 2-81 months). Preservation of the larynx in this series was accomplished in 6/8 patients (75%), and local control was obtained in 7/8 patients (87%) (Table 4). Overall 5-year survival was 50% (Figure 1).

**Table 3:** TNM classification of the initial tumor, post-radiotherapy recurrence and stage migrations after salvage surgery due to radiotherapy failure

	Stage at recurrence		Pathological stage			# Patients	Migration of stage
	N0	N+	N0	N+	Upstaged	4	T1bN0 (I)→ T2N0 (II)
			
rT1	4	0	1	0			T1aN0 (I)→ T2N0 (II)
			
rT2	4	0	5	1			T1bN0 (I)→ T2N1 (III)
			
rT3	0	0	0	1			T2N0 (II)→ T3N1 (III)

Total	8	0	6	2	Downstaged	1	T1bN0 (I) → T1aN0 (I)

rT, recurrent T stage.

**Table 4:** Literature review and comparison regarding CHEP after radiotherapy

Reference	# Cases	# Cases (CHEP)	Organ preservation (%)	DFS (%)	Overall survival
[11]	12	4	75	87	87% (3 years)

[16]	23	18	66.6	74	82.9% (3 years)

[14]	45	15	87	95.4	--

[17]	9	6	78	78 (three patients had a follow-up <3 years)	--

[18]	21	4		76	85% (3 years)

Current series	8	8	75	87	50% (5 years)

DFS, disease-free survival

## Discussion

Radiotherapy continues to be the most frequently used treatment for glottic carcinoma of the larynx in many oncological centers; however, at our institution we have recently instituted changes to this approach by more often offering surgical treatment for lesions in their early stages [13]. In this study, one of the most interesting aspects was the assessment of postradiotherapy recurrences. Total laryngectomy continues to be the most used procedure for this. This is mainly due to the lack of experience in the techniques of conservative surgery of the larynx as well as to the notion of a marked increase of complications that some surgical groups have associated with partial laryngectomies. Surgical salvage treatment with SCPL is possible in selected patients who seek medical care, presenting a similar clinical status to the initial condition and/or with progression but complying with the classical criteria established for this surgery. These are currently less limiting than their original description by Laccourreye et al. [12]. In this regard, a report of the main European group on 12 patients [11] has prompted the use of the SCPL approach for postradiotherapy recurrences in Mexico. Only one group in Mexico has published cases on partial laryngectomies in previously irradiated patients [7], although they include only two cases with SCPL but with cricohyoidopexy. Recently, results have been published from other series [14-16] with the same purpose using SCPL with CHEP.

Mean age of patients in our series was 67 years, similar to that reported by Laccourreye et al. [11]. Mean follow-up time after completion of radiotherapy was 30 months, although one patient was radiated 14 years prior. Mean follow-up after salvage surgery was 34.5 months, similar to that reported in other series [11-16]. We found no difference in either tracheostomy time or time for removal of feeding tube as compared to nonirradiated patients [13]. This is similar to the report by Spriano et al. [15] but contrary to the series by Laccourreye et al. [11] who demonstrated 2-fold longer times in previously irradiated patients. As suggested by Laccourreye et al. [11], increase in time is primarily due to the marked increase in the frequency of postoperative edema of the arytenoids and in the well-known delay in tissue healing and cicatrization. This situation occurred in our case #3 who did not respond to steroids and required surgery. However, subsequent complications are brought about by inexperience in the management of these cases in which only an incision in the edematous mucosa should be made, either with laser or other cutting material, and not extensive removal of the mucosa. This conditions a significant cicatrization procedure leading to immobility of the arytenoids. In retrospect, this is what conditioned the ankylosis of the arytenoids in our patient who then required endoscopic gastrostomy for a 2-year period due to chronic aspiration without pulmonary repercussion. During that time, he remained under phoniatric rehabilitation. After this 2-year period the patient was able to eat normally. Likewise, long-term tracheostomy and impairments in cicatrization may induce a tracheocutaneous fistula, which in our case #3 did not affect the patient's quality of life because it was only 3 mm but continues to persist.

The most severe complication is rupture of the pexy. According to our experience, it is possible to overcome this complication through a new pexy procedure. In our present case #5, however, this was not possible due to patient's age and associated comorbidities. Therefore, total laryngectomy was performed. However, in younger patients with better functional reserve, it is possible to preserve the organ by placing a Montgomery T tube, allowing for adequate cicatrization and larynx preservation.

It has frequently been reported [17-19] that the possibilities of performing partial laryngectomies in cases of recurrence after radiotherapy are conditioned to early stages or to those not progressing during therapy. This is only partially true as demonstrated in our series where we observed a migration to more advanced stages in half of our cases (Table 3). It is our opinion that patients previously subjected to radiotherapy should be treated as if they had not been previously irradiated and must comply only with the same requirements as those needed for conservative surgery. The main problem is that each conservative surgery has its own precise indications and only a few groups dominate the vast range of conservative surgeries. Finally, organ preservation in this series was 75% with local control being 87%, similar to other reports (Table 4) [11,16,18].

In conclusion, in selected patient with persistence and/or recurrence after radiotherapy due to cancer of the larynx, SCPL with CHEP seems to be feasible with acceptable local control and toxicity. Complications may be encountered as in previously nonirradiated patients; however, they may be greater because irradiated tissue is involved. Likewise, these complications must be treated conservatively to avoid altering laryngeal function, which is the objective of the surgery. As we have proposed, in every conservative surgery intra-operative assessment must be performed to determine surgical margins. Subsequent conservative treatment is not feasible, and disease-free margins must be ensured.

## Competing interests

The authors declare that they have no competing interests.

## Authors' contributions

KLO: Conception and design, data acquisition, interpretation and writing of the paper. PP: Conception and design and review of the article. ECR: Data acquisition and drafting the manuscript. MTV: Data acquisition and drafting the manuscript. VVV: Responsible for statistical analysis of the information. All authors read and approved the final manuscript.
